# Bridging technology and sustainability: examining the role of green AI adoption in Indian banking sector

**DOI:** 10.3389/frai.2025.1692763

**Published:** 2026-01-12

**Authors:** Sarath Chandran M. C., Renju Chandran, Krishnashree Achuthan

**Affiliations:** 1 Amrita School of Arts, Humanities and Commerce, Amrita Vishwa Vidyapeetham, Amritapuri Campus, Amritapuri, India; 2 School of Contemporary Knowledge Systems, Chinmaya Vishwa Vidyapeeth, Ernakulam, India; 3 Center for Cybersecurity Systems and Networks, Amrita Vishwa Vidyapeetham (Deemed to be University), Amritapuri, India

**Keywords:** green AI, emerging economies, TOE-TAM framework, sustainable banking, indian banks

## Abstract

The rapid integration of Artificial Intelligence (AI) in India’s banking sector offers operational benefits but also raises sustainability challenges. This study focuses on “Green AI,” defined as AI technologies optimized for energy efficiency and carbon conscious practices, by extending the Technology–Organization–Environment (TOE) and Technology Acceptance Model (TAM) frameworks with sustainability-linked factors. Data were collected from 412 mid- to senior-level professionals across six leading public and private banks, and Structural Equation Modeling (SEM) was employed to test the proposed hypotheses. Findings reveal that Banking Infrastructure (*β* = 0.419), Financial Investment (*β* = 0.401), and Competitive Pressure (*β* = 0.329) are the strongest predictors of Green AI adoption, while Regulatory Influence (*β* = 0.147), Perceived Usefulness (*β* = 0.129), and Perceived Ease of Use (*β* = 0.098) exert weaker but significant effects. Adoption of Green AI demonstrates a positive link to sustainability outcomes (*β* = 0.446), indicating its potential to convert structural readiness into measurable environmental gains. Although direct energy-consumption data were unavailable, perceptual measures provided valid proxies aligned with emerging-market studies. The results suggest that resource and market drivers outweigh attitudinal factors, offering actionable insights for infrastructure investment, regulatory refinement, and ESG integration, with implications for other emerging economies.

## 1 Introduction

Artificial Intelligence (AI) has emerged as a game-changing force in banking, enabling institutions to enhance operations, customer service, and decision-making (Kumar et al., 2022). Traditional AI models, however, require tremendous computational power and, therefore, are a source of power consumption and environmental degradation. As financial institutions worldwide implement AI to automate and mitigate risks, their carbon footprint and sustainability have emerged as significant concerns (Uddin et al., 2024). The concept of Green AI, focusing on energy-efficient AI models and eco-friendly computing processes, has become a necessity to ensure that banks use AI without exacerbating environmental degradation (Ridwan et al., 2024). The banking sector’s usage of AI for fraud detection, credit risk evaluation, chatbots, and algorithmic trading has expanded exponentially, requiring the reduction of the environmental footprints of AI-based financial services (Lau, 2025).

In India, public sector banks and private sector banks are adopting AI to automate banking services, improve security, and increase customer engagement. Public sector banks (PSBs) regulated by the Reserve Bank of India (RBI) play a significant role in financial inclusion and policy-based banking, whereas private sector banks (PvSBs) are also strong advocates of innovation through technology and competitive services (Iqbal and Sami, 2017). The growing emphasis on green banking has also compelled Indian banks to adopt AI solutions with environmental, social, and governance (ESG) targets (Saif-Alyousfi and Alshammari, 2025). Different international financial regulators, like the Reserve Bank of India (RBI) and the Securities and Exchange Board of India (SEBI), have introduced sustainability frameworks for financial institutions, but little research exists on how far Indian banks have adopted Green AI (Kumaran and Kamal, 2025). While AI has significantly improved operational effectiveness, empirical research examining the energy efficiency, regulatory issues, and sustainability of implementing AI in Indian banking is limited.

At the operational level, several Indian banks have already embedded AI solutions into daily banking activities. State Bank of India employs AI-based chatbots such as “SIA” to handle customer service inquiries and reduce transaction processing burdens (Othayoth and Khanna, 2025), while HDFC Bank uses AI-powered virtual assistants (EVA) and fraud detection algorithms to enhance real-time security monitoring (Kumar Dwibedi and Kumar Sahoo, 2024). ICICI Bank has deployed machine-learning tools for credit risk assessment and transaction anomaly detection (Budda et al., 2023), whereas Axis Bank utilizes AI-driven robotic process automation (RPA) across back-office operations to improve processing efficiency (Megargel et al., 2025). Emerging Green AI initiatives are increasingly linked to cloud-based data management, energy-efficient computing practices, and optimized workload allocation. For instance, major private banks have begun migrating AI operations to renewable-powered cloud platforms and low-energy data centers, while public sector banks are aligning AI procurement and digitalization strategies with government-led green banking mandates and ESG reporting requirements (Chandran et al., 2025). These developments signify that while AI deployment is already widespread, the shift toward energy-efficient and carbon-conscious AI deployment representing Green AI adoption is still uneven and evolving across the Indian banking system.

Industry reports indicate a rapid increase in AI adoption across India’s banking sector. RBI and NASSCOM estimates suggest that over 60% of leading Indian banks have deployed at least one AI-enabled operational system, covering customer engagement, fraud analytics, or credit processing, with projected AI investment growth rates exceeding 20–25% annually (Mishra et al., 2024; Biswas et al., 2024). Industry assessments further show that AI-driven customer-service chatbots now manage over 40–50% of routine customer interactions in major private banks, while machine-learning analytics support fraud-monitoring processes across nearly all large scheduled commercial banks (Manda and Nihar, 2024; Othayoth and Khanna, 2025). Cloud-based digital transformation initiatives linked to ESG modernization have also expanded sharply, with Indian banks increasing data-center consolidation and energy-efficient computing investments as part of broader sustainable banking strategies (Chilukala, 2025). These figures confirm that AI adoption in Indian banking is no longer experimental but has entered a phase of widespread institutional deployment, creating the necessary scale for evaluating its sustainability implications through the Green AI lens.

Although AI sustainability has been explored in finance, such as carbon-aware fintech tools (Olan et al., 2022) and ESG analytics (Oguntibeju et al., 2024), studies largely treat sustainability as a peripheral outcome and focus on developed markets. Few explicitly test how organizational, technological, and environmental drivers interact to influence carbon-conscious AI adoption, nor how these drivers differ between state-owned and private banks operating under resource and policy constraints of an emerging economy (Sheth et al., 2022). By embedding sustainability variables (e.g., green investment, ESG pressure) into TOE–TAM and empirically validating mediation pathways to sustainability outcomes, this work moves beyond geography to provide structural and behavioral insights absent in prior research. Moreover, the studies have not distinguished between the perspectives of state-owned and private banks toward Green AI based on their varying business models and regulatory environments. Though private sector banks are generally leaders in adopting new technology, their AI adoption agendas might not be sustainability-centric (Hidayat-ur-Rehman and Hossain, 2024). Conversely, public sector banks tend to follow policy-oriented initiatives, so their AI adoption strategy is regulatory-compliant rather than innovation-fostered (Raj and Puri, 2024).

Moreover, there is limited research on the impact of government policy and regulatory measures on the energy-efficient implementation of AI in banking. Bridging these gaps is essential in developing an all-encompassing framework that will guide banks in adopting AI technologies that are efficient, secure, and sustainable. The present study seeks to fill these gaps by conducting a comprehensive analysis of the way Indian banks are adopting Green AI and to what extent sustainability concerns propel their AI programs. The research looks into both the public and private banks, comparing their respective approaches towards adopting AI, regulatory compliance, and sustainability objectives. The study examines the challenges and opportunities of green AI adoption, assessing how regulatory regimes, cost considerations, technological infrastructure, and energy consumption affect the AI strategies of banking institutions. Further, this study aims to determine whether banks’ AI projects are in line with broader climate action policies, such as India’s Paris Agreement net-zero carbon emissions goal and the United Nations Sustainable Development Goals (SDGs).

Despite the proliferation of AI in global finance, the environmental externalities of these technologies are understudied. India’s banking sector, driven by both technological modernization and policy imperatives like SEBI’s BRSR and the RBI’s green finance guidelines, presents a crucial testbed for sustainable AI (Mishra et al., 2024; Biswas et al., 2024; Chandran, 2025). Yet, Green AI’s integration into operational and compliance frameworks remains fragmented. This study seeks to fill that gap by investigating how Indian banks are deploying Green AI to achieve dual outcomes: digital efficiency and environmental sustainability. While the findings speak to emerging market dynamics, this study is India-specific and does not include cross-country data; therefore, generalization to other economies requires caution and further empirical testing.

*RQ1*: To what extent are Indian banks leveraging Green AI to enable sustainable banking operations?

*RQ2*: What are the key differences in Green AI adoption in public sector banks and private sector banks?

*RQ3*: How is the Indian banks’ embrace of Green AI impacted by regulatory policies?

*RQ4*: What are the technological barriers to the implementation of Green AI in banking?

*RQ5*: How does Green AI adoption contribute to sustainability outcomes in Indian banking?

Unlike previous TOE–TAM studies that focus on generic IT adoption, this paper empirically extends the framework to Green AI by integrating sustainability-linked factors such as carbon-aware investments and ESG pressures into the Indian banking context. It provides first-of-its-kind evidence on how infrastructure and financial commitment dominate perceptions in driving sustainable AI adoption, and positions Green AI as a mediating construct translating these drivers into measurable environmental performance.

The present study is highly relevant to several stakeholders, including banking CEOs, policymakers, vendors of technology, and financial regulators, as it provides insights regarding the strategic embrace of Green AI in banks. By identifying the best practices, regulatory barriers, and technology gaps, the study contributes to the prevailing debate on green banking. Moreover, the research identifies how financial institutions can benchmark their AI sustainability policies against global best practices to determine compliance with local and international environmental regulations. The study’s findings also benefit government agencies and financial policymakers by helping them develop incentives, subsidies, or regulatory guidelines that promote sustainable AI adoption by the banking sector.

Moreover, the study’s contributions extend beyond banking institutions to technology developers and fintech operators that provide solutions based on AI to financial services. Understanding how effective AI models could be implemented in banking operations will enable fintech firms to design new and green AI solutions that complement the financial sector’s sustainability agenda. In addition, academic scholars engaged in the study of AI ethics, fintech, and sustainability can use this study to enhance the theoretical foundation of the Green AI role in finance. Through the provision of quantitative evidence on the impact of Green AI in banking, the research provides a map for harmonizing sustainable strategies with AI, resulting in double bottom-line improvement in finance and the environment.

This study offers threefold contributions: (1) Theoretically, it extends the TOE-TAM framework to a novel green AI context by integrating disaggregated predictors with sustainability-linked outcomes. (2) Empirically, it applies SEM-based mediation analysis to reveal how banking infrastructure and investment influence carbon-conscious AI adoption. (3) Practically, it provides regulators and bank managers with actionable policy recommendations aligned with India’s Net Zero 2070 commitments.

The structure of this paper is in a sequential order to provide a thorough comprehension of Green AI adoption in Indian banking. Section two provides a detailed literature review, which gives an overview of existing studies on AI in banking, sustainability concerns, and regulatory environments impacting AI adoption. The third section: research methodology, outlines the research design, data collection process, and analysis. Section four deals with results: provides empirical evidence, presenting key statistical results on the impact of Green AI in banking operations. This is succeeded by discussion section, where results are interpreted in terms of banking sustainability and policy implications. Finally, the conclusion section summarizes key findings, highlights study limitations, and suggests avenues for future research.

## 2 Literature review and hypothesis formulation

Green AI marks a shift from performance-focused AI to environmentally optimized AI models, guided by sustainability principles and frameworks such as the Triple Bottom Line (Elkington, 2004) and ESG scoring. Key outcomes include energy efficiency, regulatory compliance, and reputational gains. However, few studies in emerging markets, especially India, have empirically linked Green AI adoption to these multidimensional outcomes across public and private banks. Previous studies emphasized AI’s role in fraud detection, credit risk, customer service, and algorithmic trading (Olan et al., 2022; Sheth et al., 2022; Oguntibeju et al., 2024; Kaur et al., 2020; Boulieris et al., 2023). These works highlight operational benefits but overlook energy consumption and environmental implications (Khalid, 2024). In response, Green AI is emerging as an alternative, promoting energy-efficient architectures and carbon-aware designs (Bolón-Canedo et al., 2024; Barbierato and Gatti, 2024; Olawade et al., 2024). While some developed-economy institutions have adopted carbon-aware computing and AI-driven sustainability monitoring (Rathor et al., 2024), adoption in emerging markets like India remains largely unexplored.

Regulators such as RBI, SEBI, and the FSB have introduced sustainability-linked policies (Bharanitharan and Kaur, 2024), but AI-specific environmental guidelines are nascent. RBI’s Green Finance Initiatives promote carbon monitoring tools and ESG-compliant banking (Nicoletti, 2021; MC et al., 2025), yet policies focus more on ethics and privacy than sustainability (Shobanke et al., 2025). The absence of enforceable environmental regulations creates uncertainty for banks. Public sector banks (PSBs) often follow government-mandated agendas, but legacy infrastructure, high costs, and low investment constrain innovation (Vedapradha Radhakrishna et al., 2024). Private banks (PVSBs) like HDFC, ICICI, and Axis have pursued more aggressive digital transformation (Malhotra et al., 2025), yet the extent of true Green AI strategies remains unclear (Jain, 2024), also comparative evidence on public–private differences are scarce (Rahman et al., 2023).

The costs of shifting to energy-efficient AI (hardware upgrades, renewable-powered data centers) are high (Farzaneh et al., 2021; Awogbemi et al., 2024). Data security and transparency add complexity (Díaz-Rodríguez et al., 2023; Vijayagopal et al., 2024). Few studies use quantitative techniques to assess Green AI performance (Mondal et al., 2024). SEM-based studies (Patel et al., 2021) suggest potential for more robust causal insights, but adoption and sustainability effects remain under-measured. Unlike prior TOE–TAM studies focused on generic IT adoption, this paper contextualizes the framework for sustainable digitalization by (i) re-specifying predictors such as financial investment for carbon-aware infrastructure and competitive ESG pressure, (ii) operationalizing Green AI adoption as a mediating construct, and (iii) linking these drivers to sustainability outcomes. This disaggregated design adapts established constructs to a distinct environmental-technology domain. Moreover, it introduces a disaggregated assessment of Sustainability Outcomes (SO) (energy efficiency, ESG compliance, carbon footprint) to offer clearer practical insights for banks and regulators. This multidimensional approach addresses critical gaps in both the AI adoption and green finance literature (Chandran and Sarath Chandran, 2024).

### 2.1 Theoretical background and conceptual framework

The present study is anchored in the Technology–Organization–Environment (TOE) framework, augmented by Perceived Usefulness (PU) and Perceived Ease of Use (PEOU) from the Technology Acceptance Model (TAM) (Oliveira and Martins, 2011; Ifinedo, 2012). This integration captures both structural drivers and decision-makers’ perceptions relevant to Green AI adoption in Indian banking.

Beyond standard TAM variables, the model emphasizes resource-intensive infrastructure, budgetary commitment to energy-efficient AI, and ESG-related pressures not previously combined. TOE–TAM is extended beyond technology acceptance to environmental performance, with Green AI adoption treated as a mediator translating these drivers into measurable sustainability outcomes. Following calls for granularity in emerging market studies (Baker, 2012; Ramdani et al., 2009), each TOE dimension is assessed at the factor level: PU, PEOU (technology); Financial Investment (FI), Banking Infrastructure (BI) (organization); Regulatory Influence (RI), Competitive Pressure (CP) (environment). This enables testing of individual drivers rather than broad latent blocks, offering richer theoretical and practical insights.

‘Green AI’ here refers to AI systems designed, deployed, and governed with environmental sustainability in mind (Alzoubi and Mishra, 2024; Barbierato and Gatti, 2024). It includes carbon-conscious algorithm design, energy-efficient hardware, and data centers, and AI-enabled ESG monitoring (Mei et al., 2024). This study measures green AI adoption via three perceptual indicators: policy integration, impact reduction, and strategic prioritization, validated through expert consultation in Indian banking. Although direct technical measures (e.g., energy logs) were unavailable, perceptual proxies are widely accepted in organizational IT research.

**Figure 1:**
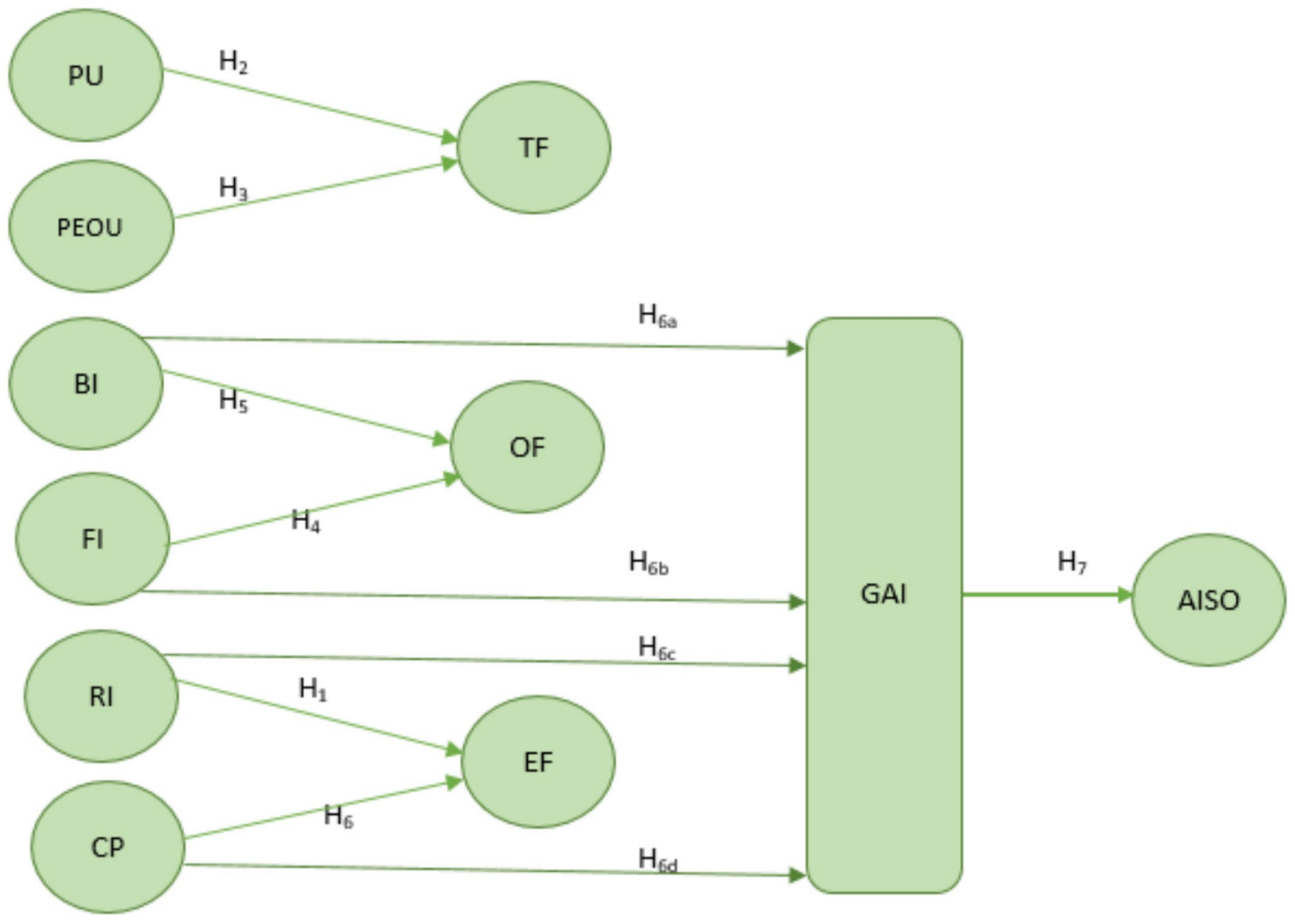
Disaggregated conceptual framework based on TOE-TAM Model. The model reflects both direct effects of TOE-TAM constructs on Green AI Adoption (GAI) and indirect effects on Sustainability Outcomes (SO) through GAI, consistent with the mediation paths validated in the structural model. PU, Perceived Usefulness; PEOU, Perceived Ease of Use; FI, Financial Investment; BI, Banking Infrastructure; RI, Regulatory Influence; CP, Competitive Pressure; TF, Technological Factors; OF, Organizational Factors; EF, Environmental Factors; GAI, Green AI Adoption; SO, Sustainability Outcomes.

Conventional AI adoption research has focused on efficiency, automation, and risk. This paper reframes AI as a dual-purpose innovation, aligning modernization with energy efficiency and ESG goals (Jing and Zhang, 2024). By empirically validating Green AI as a mediating construct using disaggregated TOE–TAM predictors, this study advances Green FinTech literature and addresses key gaps in AI adoption research for emerging economies. Each factor is hypothesized to influence Green AI adoption (GAI), which in turn drives sustainability outcomes (SO) operationalized as energy efficiency, ESG compliance, and carbon footprint reduction. Figure 1 depicts the conceptual model based on the theoretical background.

#### 2.1.1 Regulatory influence and green AI adoption

Banks function under a regulated environment, where government policies, compliance requirements, and green banking frameworks have a crucial role in the adoption of AI (Singh, 2022). Regulators like the RBI and SEBI implement sustainability strategies that push banks to adopt AI-based sustainability processes. As institutional compliance and green environment have emerged as regulatory priorities, Green AI adoption is expected to be positively influenced by regulatory pressure and policy mandates (Akhtar et al., 2024). Hence, we hypothesize that:

*H_1_*: Regulatory influence has a positive impact on Green AI adoption in the banking sector.

#### 2.1.2 Perceived usefulness and green AI adoption

The technological dimension of the TOE framework suggests that banks are more likely to adopt Green AI if it is perceived as enhancing energy efficiency, reducing operational costs, and improving decision-making (Mohammad et al., 2022). AI-driven sustainability tools enable banks to minimize computational energy consumption, automate risk management, and streamline financial transactions, which can significantly enhance operational efficiency.

*H_2_*: Perceived usefulness has a positive impact on Green AI adoption in the banking sector.

#### 2.1.3 Perceived ease of use and green AI adoption

The ease of implementation of AI-based sustainability solutions by banks will decide how much they are willing to embrace the technology (Kaur et al., 2024). If Green AI is complex to implement, complicated, or not compatible with existing banking systems, its adoption will be low (Akhtar et al., 2024). However, if AI-based sustainability models are streamlined and can be easily integrated into banking infrastructure, banks will be more inclined to use them.

*H_3_*: Perceived ease of use has a positive impact on Green AI adoption in the banking sector.

#### 2.1.4 Financial investment and green AI adoption

The organizational dimension of the TOE framework indicates that financial investment is essential for technology adoption (Al Hadwer et al., 2021). The use of AI-based sustainable banking solutions entails capital spending on computing hardware, AI model fine-tuning, and regulatory compliance (Alzoubi and Mishra, 2024). Banks with more financial resources will be better placed to adopt Green AI compared to banks with limited investment capacity. Therefore, we hypothesize that:

*H_4_*: Financial investment has a positive impact on Green AI adoption in the banking sector.

#### 2.1.5 Banking infrastructure and green AI adoption

A bank’s technological and digital infrastructure significantly influences its ability to integrate AI-based sustainability solutions. Banks that have updated IT systems, cloud computing systems, and AI-based risk management systems are better positioned to adopt Green AI in their business models (Alqahtani et al., 2024). Conversely, banks with outdated IT systems and legacy infrastructure may face technological barriers to adoption (Saxena et al., 2024).

*H_5_*: Banking infrastructure has a positive impact on Green AI adoption in the banking sector.

#### 2.1.6 Competitive pressure and green AI adoption

The environmental factor of TOE implies that peer pressure and competition in the market play a crucial role in the adoption of technology (Chittipaka et al., 2022). Indian banks are increasingly under pressure to align with global sustainability benchmarks and enhance their Environmental, Social, and Governance (ESG) performance. Competitive forces compel financial institutions to embrace sustainable AI practices so that they do not become outdated in a rapidly evolving digital banking age (Kumaran and Kamal, 2025).

*H_6_*: Competitive pressure has a positive impact on Green AI adoption in the banking sector.

Green AI is expected to serve as a critical enabler of sustainability within banking institutions by transforming technological, organizational, and environmental inputs into sustainable outcomes. According to TOE and TAM logic, external drivers (e.g., infrastructure, investment, pressure) may not directly result in sustainability unless they foster actual Green AI deployment. Hence, we hypothesize:

*H6_a_*: Green AI Adoption mediates the relationship between Banking Infrastructure and Sustainability Outcomes.

*H6_b_*: Green AI Adoption mediates the relationship between Financial Investment and Sustainability Outcomes.

*H6_c_*: Green AI Adoption mediates the relationship between Competitive Pressure and Sustainability Outcomes.

*H6_d_*: Green AI Adoption mediates the relationship between Regulatory Influence and Sustainability Outcomes.

#### 2.1.7 Green AI adoption and sustainability outcomes

The relationship between Green AI adoption and sustainability outcomes is the core theme of the study, which examines how integrating AI-based sustainable solutions pays off in measurable banking process improvements (Kulkov et al., 2023). Banks successfully implementing Green AI technologies are likely to reap the rewards of enhanced energy efficiency, reduced operating costs, and improved environmental regulatory compliance (Chen et al., 2023). Additionally, adopting sustainable AI practices can advance the corporate social responsibility (CSR) initiatives of a bank, potentially contributing to enhanced consumer trust and investor confidence. By connecting AI-driven strategy with ESG considerations, banks can advance their sustainability performance while being technologically competitive within the financial sector (Bansal et al., 2024).

*H_7_*: Green AI adoption has a positive impact on sustainability outcomes in the banking sector.

Although the TOE model theoretically supports a mediating effect of Green AI Adoption (GAI) between contextual factors and Sustainability Outcomes (SO), we restricted our analysis to direct effects due to the cross-sectional nature of the data. Mediation analysis may be better suited for future longitudinal studies. Future studies could conduct and test these mediating pathways through longitudinal or multi-wave data, using bootstrapped mediation models to demonstrate how organizational and environmental readiness translates into sustainable outcomes via AI adoption.

## 3 Materials and methods

The study follows a quantitative research design to explore the factors of Green AI adoption among Indian banks (Sharma et al., 2024). A cross-sectional survey research design is employed, which allows for data collection specifically from AI adoption, regulatory compliance, and sustainability practices. The research is grounded on the TOE framework, systematically investigating why technological, organizational, and environmental factors give rise to Green AI adoption. Since the study aims to test causal relationships between sustainability outcomes and Green AI adoption, Structural Equation Modeling (SEM) is utilized as the primary analysis method. SEM is ideal for confirming theoretical models and estimating the inter-relationships between more than one construct (Shrestha et al., 2023).

The primary data collection utilized a standard questionnaire to gather responses for core variables in the TOE model. The questionnaire consists of closed-ended Likert-scale items, which offer a standardized and measurable assessment of variables such as regulatory influence, technological feasibility, and competitive pressures. By using this structured approach, reliability, objectivity, and comparability across responses are achieved. A complete list of the survey items used for each construct is provided in Appendix Table 1A, offering transparency regarding construct operationalization and adherence to established measurement standards.

### 3.1 Sampling and data collection procedure

The study targeted mid- to senior-level professionals directly engaged in AI strategy, technology management, compliance, and sustainability within banks. Respondents included executives, IT strategists, AI implementation managers, and sustainability officers, each required to have prior experience in AI-based banking operations and sustainability initiatives. Screening questions ensured respondents had decision-making or oversight responsibilities, enhancing the validity of their inputs (Rahman et al., 2021).

Invitations were distributed to professionals from six leading Indian banks – State Bank of India (SBI), Punjab National Bank (PNB), HDFC Bank, ICICI Bank, Axis Bank, and Kotak Mahindra Bank – representing both public and private sectors. These six institutions were purposively selected because they collectively represent a substantial share of India’s formal banking market and operational diversity. SBI and PNB are the two largest public sector banks by asset base and customer outreach, jointly accounting for a major proportion of government-mandated financial inclusion activity, branch networks, and legacy banking infrastructure (Gupta et al., 2023). HDFC Bank, ICICI Bank, Axis Bank, and Kotak Mahindra Bank represent the largest and most technologically advanced private-sector banks, consistently recognized as frontrunners in digital transformation and fintech adoption (Sneh et al., 2024). These banks cover a dominant segment of retail, corporate, and digital banking transactions in India and serve as representative pioneer of AI diffusion across both regulated public banking systems and competitive private banking environments. While the sample does not include smaller regional or cooperative banks, the selected institutions are widely considered as trend-setters whose technological practices subsequently shape sector-wide adoption patterns. Surveys were administered via verified LinkedIn banking groups, formal industry networks, and direct corporate contacts over an 8–12-week period (July–September 2024). Screening questions confirmed respondents’ roles and experience to minimize bias.

A total of 412 valid responses were obtained from 615 invitations (67% response), exceeding the recommended 10:1 observation-to-variable ratio for SEM and aligning with previous AI adoption studies (200–400 respondents). Although the conceptual model is at the organizational level, respondents’ views were used as perceptual proxies for institutional behavior, a common practice in TOE-based studies (Ifinedo, 2012; Ramdani et al., 2009). Where possible, multiple respondents were collected from the same bank; where only one was available, the individual’s managerial perspective was retained. While we did not compute inter-rater reliability, the emphasis on senior roles, screening, and sectoral diversity helps reduce bias. Future studies could expand by validating aggregation through the Intraclass Correlation Coefficient (ICC) or similar metrics.

### 3.2 Constructs

The questionnaire was developed using validated scales from previous studies and reviewed by banking professionals and academic researchers in fintech and sustainability. A pilot test with 30 respondents ensured clarity, reliability, and content validity. The final instrument included 30 items across nine sections (demographics, RI, PU, PEOU, FI, BI, CP, GAI, SO), measured on a 5-point Likert scale (1 = strongly disagree, 5 = strongly agree). Reliability and validity were assessed using Cronbach’s alpha, Composite Reliability (CR), Average Variance Extracted (AVE), and Confirmatory Factor Analysis (CFA) in AMOS v.29 (Maximum Likelihood Estimation). Model fit was evaluated using CFI, RMSEA, SRMR, and χ^2^/d.f. To reduce potential common method bias, procedural remedies were applied, including assured anonymity, screening for decision-making roles, and randomizing items. A post-hoc Harman’s single-factor test indicated that no single factor accounted for a majority of variance (largest factor < 40%), suggesting common method bias (CMB) was not a major concern. CFA results further confirmed model fit and discriminant validity, strengthening confidence in the measures.

Data analysis was conducted with Smart-PLS and AMOS v.29 for SEM-based hypothesis testing. GAI was measured using a 30-item instrument (Al-Khatib, 2023). RI was assessed using 4-item scales (Brown et al., 2006; Oyeniyi et al., 2024); PU (five items, Yun et al., 2021); PEOU (four items, Davis, 1989); FI and BI (four items each, Zhu et al., 2023; Rahman et al., 2023); CP, GAI, and SO (three items each, Emmanuel et al., 2024; Mei et al., 2024; Rahman et al., 2023). SO reflected organizational efforts to integrate AI into sustainability policies, reduce environmental impact, and prioritize sustainable development. Although technical indicators (e.g., AI energy audits, carbon metrics) were unavailable, validated perceptual items served as organizational proxies, consistent with Green FinTech research (Alzoubi and Mishra, 2024). This perceptual approach is justified by the novelty of GAI adoption in emerging markets and limited organizational data availability.

## 4 Results

### 4.1 Demographical information

Table 1 presents the demographic profile of the respondents. The gender distribution shows 58.7% male (*n* = 242) and 41.3% female (*n* = 170), reflecting a balanced participation rate. The age group of 25–45 years is the largest (66.8%), which implies that younger professionals are more active in AI-based sustainability efforts. Regarding education, 43.0% are postgraduates, 40.3% are undergraduates, and 11.9% possess doctorates, reflecting that AI adoption choices are made by highly educated professionals. Based on their job positions, 22.19% are Senior Managers, 21.42% are Managers for AI & Compliance, 21.41% are Sustainability Officers, 20.4% are Assistant General Managers, and 15.1% are IT Officers. The dominance of the managers and sustainability officers reflects that AI-driven sustainability activities are made by mid-to-senior-level professionals with decision-making powers.

**Table 1:** Profile of respondents.

Demography	Category	Frequency
Gender	Male	242
	Female	170
Age	25–35 years	142
	36–45 years	133
	46–55 years	70
	56 & above	67
Education	UG	166
	PG	177
	Ph.D.	49
	Others	20
Job Position	Assistant General Manager	85
	IT Officer	60
	Manager (AI & Compliance)	88
	Senior Manager	91
	Sustainability Monitoring Officer	88
		*N* = 412

Source: Author’s source (*n* = 412).

### 4.2 Confirmatory factor analysis and measurement model estimation

The data in Table 2 provided evidence for the measurement model’s validity and reliability. To validate the measurement model, Confirmatory Factor Analysis (CFA) was performed. The results confirmed good construct reliability and convergent validity, as shown in Table 2. Composite Reliability (CR) values exceeded 0.70 for all constructs, and the Average Variance Extracted (AVE) values were all above 0.50, indicating strong convergent validity. The CFA model fit indices presented in Table 3 (CFI = 0.961, RMSEA = 0.043, SRMR = 0.042) confirm the robustness of the measurement model (Hair et al., 2019).

**Table 2:** Model validity.

Constructs	CR	AVE	MSV	MaxR(H)	*p*
RI	0.914	0.817	0.667	0.915	<0.001
PU	0.947	0.738	0.564	0.950	
PEOU	0.953	0.771	0.604	0.959	
FI	0.968	0.802	0.643	0.971	
BI	0.942	0.807	0.651	0.943	
CP	0.976	0.701	0.611	0.978	
GAI	0.972	0.819	0.645	0.979	
SO	0.975	0.829	0.596	0.977	

Source: Author calculation.

**Table 3:** Model fit.

Fit index	Values	Threshold limit	Interpretation
*χ*^2^	275.160	–	–
D.F.	204	–	–
*χ*^2^/df (Normed *χ*^2^)	1.236	1–3	Satisfied
CFI	0.961	≥ 0.95	Satisfied
SRMR	0.042	≤ 0.08	Satisfied
RMSEA	0.043	≤ 0.06	Satisfied
PClose (RMSEA Close Fit Test)	0.894	> 0.05	Satisfied

Source: Author calculation.

Convergent validity (all CR > 0.70, AVE > 0.50, loadings > 0.70; see Appendix Table 2A) and discriminant validity (Fornell–Larcker; HTMT < 0.90) were satisfied (see Appendix Table 2B) (Fornell and Larcker, 1981; Henseler et al., 2015).

The construct SO was operationalized as a second-order latent variable combining perceived outcomes related to energy efficiency, carbon footprint reduction, and ESG compliance. While these are conceptually distinct, EFA and CFA revealed high inter-item correlations and factor loadings above 0.80, supporting the one-dimensionality of the aggregated construct. AVE of 0.829 and CR of 0.975 confirm convergent validity. Nevertheless, we acknowledge the importance of decomposing SO into specific outcome constructs in future research for more granular analysis.

The model fit indices depicted in Table 3 indicate a strong fit between the measurement model and the data. The χ^2^ value (275.610, df = 204) produced a χ^2^/df of 1.236, suggesting a satisfactory model fit. CFI (0.961), SRMR (0.042), and RMSEA (0.043) met the recommended thresholds, confirming good incremental and residual fit. The PClose value of 0.894 indicates a close model fit. The results confirm that the measurement model is a good fit and appropriate for further SEM analysis.

### 4.3 Structural model

The efficiency of the independent constructs in predicting the variability of the dependent construct was assessed using the structural model analysis (Albahri et al., 2021). The structural model utilized in this study is depicted in Table 4. The suggested hypothesis was examined using SEM in SmartPLS. Three components were used to make up the model: TOE, i.e., (R1-R4, PU1-PU4, PEOU1-PEOU5, F1-F4, BI1-BI4, CP1-CP3, GAI1-GAI3, SO1-SO3) as independent variables, and GAI and SO as dependent variables. Table 4 shows the results of path analysis.

**Table 4:** Estimates to unstandardised estimates.

Structural path	Estimates	S.E.	C.R.	*p*
GAI ← RI	0.431	0.058	7.531	<0.001
GAI ← PU	0.395	0.054	7.315	
GAI ← PEOU	0.223	0.056	7.112	
GAI ← CP	0.160	0.045	7.234	
GAI ← FI	0.366	0.061	7.610	
GAI ← BI	0.418	0.051	8.361	
SO ← GAI	0.467	0.053	8.831	

Source: Author calculation.

Considering the Table 4 unstandardized estimates and Table 5 standardized coefficients, all hypothesized paths were statistically significant (*p* < 0.001). Banking Infrastructure (*β* = 0.419), Financial Investment (*β* = 0.401), and Competitive Pressure (*β* = 0.329) show the largest standardized effects on Green AI adoption, whereas Regulatory Influence (*β* = 0.147), Perceived Usefulness (*β* = 0.129), and Perceived Ease of Use (*β* = 0.098) are significant but comparatively small. Green AI adoption → Sustainability Outcomes is positive and sizable (*β* = 0.446). These results indicate that structural and resource-based controls dominate belief-based drivers in this sample.

**Table 5:** Validation of hypothesis result.

Hypothesis	Regression weight (standardized *β*)	Results	Conclusion
H1	0.147	RI is positively related to GAI	Accepted
H2	0.129	PU is positively related to GAI	Accepted
H3	0.098	PEOU is positively related to GAI	Accepted
H4	0.329	CP is positively related to GAI	Accepted
H5	0.401	FI is positively related to GAI	Accepted
H6	0.419	BI is positively related to GAI	Accepted
H7	0.446	GAI is positively related to SO	Accepted

Source: Author calculation.

### 4.4 Hypothesis result

The significance of each structural path was tested by the bootstrapping technique at 95% sig. Level. As shown in Table 5, all the proposed hypotheses (H1–H7) were confirmed at *p* < 0.001. In particular, Banking Infrastructure (*β* = 0.419), Financial Investment (*β* = 0.401), and Competitive Pressure (*β* = 0.329) indicated strong positive correlations with Green AI Adoption. The other predictors, such as Regulatory Influence (*β* = 0.147), Perceived Usefulness (*β* = 0.129), and Perceived Ease of Use (*β* = 0.098), also showed significant but comparatively lower effects. Moreover, Green AI Adoption had a significant impact on Sustainability Outcomes (β = 0.446), which supports H7. It supports the model and emphasizes the influence of both environmental and organizational drivers on sustainable AI adoption in Indian banking.

Further, to assess the mediating role of GAI adoption, bootstrapping was conducted in SmartPLS. The results (Table 6) demonstrate significant indirect effects from the TOE predictors BI, FI, CP, and RI to SO through GAI. This confirms the partial mediation role of GAI and supports hypotheses H6a to H6d.

**Table 6:** Mediation effects of green AI adoption.

Hypothesis	Path	Indirect effect	*t*-value	*p*-value	Result
H6a	BI → GAI → SO	0.132	3.42	<0.001	Supported
H6b	FI → GAI → SO	0.148	3.89	<0.001	Supported
H6c	CP → GAI → SO	0.116	2.74	0.006	Supported
H6d	RI → GAI → SO	0.124	2.93	0.004	Supported

Source: Author calculation.

Mediation effects were tested using bootstrap resampling (5,000 iterations) on the existing sample (*n* = 412). All indirect effects were statistically significant at *p* < 0.01.

### 4.5 Endogeneity considerations

Endogeneity was a potential concern given the non-experimental, cross-sectional design and the possible reciprocal influence of constructs like FI and BI on GAI. To address this, we followed recent methodological guidance (Antonakis et al., 2010) and tested for latent endogeneity using a Gaussian Copula correction, which is suitable for SEM settings with non-linear effects (Park and Gupta, 2012). Copula terms were generated for each predictor and added to the structural model. As shown in Appendix Table 2C, none of the copula terms were significant (all *p* > 0.25), indicating that bias from endogeneity is unlikely in our estimates. Although this strengthens confidence in the results, future studies with longitudinal or instrumental-variable designs could offer even stronger causal claims.

## 5 Discussion

This study confirms that organizational and resource capabilities are the primary enablers of Green AI adoption in Indian banks. TOE–TAM integration shows that infrastructure, investment, and market signaling dominate individual attitudes, with regulation and perceptions playing a smaller role. This reflects an environment where structural readiness and competitive pressures are more immediate than policy mandates or user beliefs.

These patterns resonate with findings from other emerging economies, but also diverge in important ways from evidence in advanced markets. For instance, Udeagha and Ngepah (2023) show that in BRICS countries, financial depth, fintech capability and institutional readiness are stronger predictors of environmental performance than formal green regulations, which is consistent with our result that BI, FI and CP outweigh RI explaining Green AI adoption (Yang et al., 2025). Similarly, Rahman et al. (2021) finds that in South Asian banking, technology adoption is primarily driven by organizational readiness and competitive dynamics rather than user attitudes alone. In contrast, studies of sustainable banking services in European settings emphasize technological literacy and perceived usefulness as principal drivers of AI-enabled sustainability solutions (Fundira et al., 2024; Mei et al., 2024). Therefore, the study’s finding positions India closer to the broader emerging-economy pattern where capacity and market forces drive sustainable digitalization, while also underscoring how the relatively soft nature of domestic AI sustainability regulation keeps the effect of Regulatory Influence modest.

### 5.1 Theoretical implications

The findings deepen the TOE–TAM discourse by showing that capacity, not cognition, drives sustainability-oriented AI adoption in emerging markets. This reinforces institutional theory: banks respond to structural legitimacy and resource availability rather than perceived usefulness or ease. The weaker roles of RI, PU, and PEOU highlight that policy guidance and perceptions are enabling conditions but not decisive triggers. Green AI adoption also links directly to sustainability outcomes, confirming that environmentally optimized digital tools translate investments into measurable ESG-oriented performance. Although mediation effects were tested, they should be read as associations, not causal paths, given the cross-sectional design. These results align with recent work showing that organizational readiness outpaces regulatory and perceptual factors in shaping technology adoption (Attah et al., 2024; Udeagha and Ngepah, 2023).

Beyond emerging markets, our results also connect to a growing international literature on AI and sustainability. Kulkov et al. (2023) highlight that organizations invest in dedicated AI capabilities and process innovation are more likely to convert digitalization into progress on the Sustainable Development Goals (SDG’s), resonating our finding that BI and FI exert the strongest influence on Green AI adoption and its link to Sustainability Outcomes. Mondal et al. (2024) show, using a PLS-SEM and fsQCA approach in emerging economies, that AI contributes to net-zero transitions primarily when supported by adequate digital inclusion and financial commitment, again pointing to resource-based enablers. In contrast Jing and Zhang (2024) reported that, in Chinese manufacturing firms, AI improves ESG performance mainly through ambidextrous green innovation, with managerial perceptions of AI’s strategic value playing a more central role. Taken together, these comparisons indicate that our Indian banking evidence reinforces TOE–TAM arguments that organizational readiness and competitive pressure are the primary drivers of sustainable AI adoption. However, studies from more mature regulatory environments suggest that user perceptions and regulatory forces become more influential when clear Green AI standards and innovation-oriented governance frameworks are already in place (Bin-Nashwan and Li, 2025).

Regulatory effects remain modest, likely due to India’s lack of enforceable AI sustainability standards. RBI and SEBI guidelines exist, but they are broad and voluntary, contrasting with the EU and Singapore, where targeted frameworks strengthen compliance (Damodaran, 2023). This suggests that policy evolution could make regulation a stronger lever, while banks should not wait for mandates but invest proactively.

### 5.2 Policy and managerial implications

The results have direct implications for how Indian banks and regulators can align digital transformation with climate commitments such as India’s Net Zero 2070 target and the National Green Finance Policy. Green AI adoption can serve as a bridging mechanism between existing RBI and SEBI sustainability guidance such as climate risk expectations and BRSR disclosures and the operational realities of AI-driven banking (Chaturvedi et al., 2024).

It is important to interpret these policy implications in light of the study’s measurement approach. Direct organizational data on AI-specific energy consumption, carbon emissions, and computing workload intensity remain largely inaccessible in the Indian banking sector because present RBI and SEBI sustainability frameworks (including BRSR) do not mandate AI-level energy reporting or standardized digital-carbon disclosure. Consequently, the present study relied on perceptual proxy measures that capture managerial assessments of policy integration, operational impact reduction, and strategic prioritization of sustainable AI practices. Such perceptual indicators are widely employed in organizational technology-adoption research when objective performance metrics are unavailable, and they remain particularly appropriate in emerging regulatory environments where formal reporting systems are still evolving. Most importantly, these proxies reflect the decision-makers’ behavioral and strategic intentions that ultimately drive technology investment and implementation, making them a valid basis for deriving managerial and policy guidance under current Indian regulatory conditions.

From a managerial perspective, bank executives should prioritize three operational actions. First, they should allocate dedicated capital budgets for energy-efficient AI infrastructure, including cloud optimization, low-power data centers, and hardware refresh strategies that reduce the energy intensity of AI workloads. Second, they should establish internal Green AI governance frameworks that integrate ESG objectives into technology procurement, vendor evaluation, and model deployment, ensuring that new AI projects are assessed not only for financial returns but also for energy and carbon impacts. Third, management should create cross-functional task forces linking IT, ESG, compliance, and risk teams to monitor the carbon footprint and sustainability performance of AI systems on an ongoing basis. In public sector banks, these steps need to be accompanied by capacity-building programmes that upgrade staff skills in sustainable AI implementation (Guang-Wen and Siddik, 2022).

From a policy standpoint, the comparatively modest effect of Regulatory Influence in our model suggests that India’s current sustainability guidance for banks is necessary but not yet sufficient to shape Green AI behavior. Regulators could strengthen adoption momentum by introducing sector-specific Green AI instruments such as: (i) mandatory or “comply-or-explain” energy audits for AI operations; (ii) explicit AI-related carbon disclosure lines within SEBI’s BRSR framework; (iii) standardized Green AI procurement and data centre efficiency benchmarks for public sector banks; and (iv) regulatory sandboxes that encourage experimentation with low-energy AI architectures and carbon-aware computing. These measures would gradually move the system from voluntary ESG signaling towards measurable, AI-enabled climate accountability.

Finally, the results support a Triple Bottom Line view in which Green AI can simultaneously lower operational emissions, improve process efficiency, and enhance reputational value among investors and customers (Guang-Wen and Siddik, 2022). However, given the cross-sectional nature of the data, these implications should be interpreted as directional guidance rather than definitive causal claims, and future longitudinal work could test how sustained Green AI investments translate into concrete improvements in ESG scores and climate-risk resilience over time.

## 6 Conclusion

As banks navigate the dual pressures of digitalization and going green, Green AI comes as the strategic horizon of sustainable banking. Drawing from the TOE framework, this study derives empirical evidence on the forces driving the adoption of Green AI and its subsequent impact on sustainability outcomes within the Indian banking sector. The findings indicate that besides perceived usefulness or ease of use, the strength of a bank’s infrastructure, investment in finance, and responsiveness to competition play the most significant roles in transitioning towards AI-enabled sustainability.

These findings hold actionable significance for both banking practitioners and regulatory authorities. From a managerial standpoint, Green AI must be reframed not merely as a technological enhancement but as a strategic instrument for sustainability governance. Its integration demands alignment between IT infrastructure, ESG targets, and internal compliance mechanisms. Banks with robust digital infrastructure and capital readiness are better positioned to operationalize carbon-conscious computing, adopt energy-efficient AI architectures, and build internal capabilities to monitor AI-driven sustainability performance. Cross-functional coordination and ESG-informed AI deployment strategies are now essential to delivering both environmental and financial returns.

From a regulatory perspective, our findings expose the need for India to design sector-specific sustainability mandates for AI systems. Dedicated policies on AI energy audits, carbon scoring for digital services, and Green AI taxonomies would elevate regulatory influence and accelerate adoption. These steps would also enhance India’s compliance with the UN SDGs and the Paris Agreement. While frameworks like SEBI’s BRSR and RBI’s climate finance guidance set broad expectations, they lack specificity on AI-enabled sustainability transitions. Dedicated regulatory mechanisms are needed to focus on carbon-aware algorithm design, green AI infrastructure norms, and model transparency standards. Regulatory sandboxes, taxonomies, or compliance incentives tailored to Green AI adoption can further stimulate institutional transformation, especially in public sector banks constrained by legacy systems. Embedding Green AI into India’s broader green finance strategy would ensure that digital innovation also supports national climate goals.

### 6.1 Limitations and future research

Despite a strong methodological design, this study has several limitations. First, the findings are geographically limited to India and are based on perceptual responses from banking professionals. Although the paper references emerging market contexts, it does not include comparative data across economies; thus, generalizability to other regions remains tentative. Second, the study focuses solely on the financial services sector, particularly banking, although Green AI is also emerging in insurance, energy, logistics, and manufacturing. Future research could explore cross-sector applications, compare adoption patterns across regions (e.g., South Asia vs. Europe), and analyze longitudinal trends in AI-driven sustainability performance.

Another limitation is the aggregation of distinct sustainability outcomes into a single latent construct (SO). While this was justified through internal consistency and convergent validity, future studies could disaggregate these outcomes to examine differential effects on ESG metrics, carbon reduction, and energy efficiency. The study’s cross-sectional design also limits causal inference; longitudinal or experimental designs could capture temporal dynamics and stronger cause-and-effect evidence.

To enrich explanatory power, future work should integrate variables such as AI governance maturity, carbon accountability measures, and sector specific ESG indicators. For regulators, the findings highlight the need for actionable, India-specific frameworks. While RBI and SEBI have issued broad ESG and climate guidance, neither currently mandates AI-related sustainability metrics. Future regulatory initiatives could include: (i) an RBI-issued green AI code of practice for banks (covering energy benchmarking and AI system audits); (ii) SEBI expanding the BRSR framework to capture AI-related ESG disclosures; and (iii) a sector-wide taxonomy for AI energy and carbon reporting, modeled on the EU taxonomy but adapted to India’s market and infrastructure. Finally, mediation pathways were interpreted at the perceptual level. Future studies should incorporate multi-level or time-series data and triangulate perceptual measures with technical metrics such as AI energy consumption logs, carbon tracking, and ESG scores to strengthen the validity of sustainability claims.

## Data Availability

The raw data supporting the conclusions of this article will be made available by the authors, without undue reservation.
