# Supplementary material for: Bridging technology and sustainability: examining the role of green AI adoption in Indian banking sector
Source: Front Artif Intell. 2026 Jan 12;8:1692763. doi: 10.3389/frai.2025.1692763 (PMC12833219; doi:10.3389/frai.2025.1692763)
Supplement: Supplementary file 1 [file Data_Sheet_1.docx]

**Appendix**

Table 1A - Questionnaire

| For Demographic Information (refer to Table 1) | | |
| --- | --- | --- |
| Level of Awareness and Agreement towards the following statements.  Please indicate your level of agreement using the scale below: 5 – Strongly Agree, 4 – Agree, 3 – Neutral, 2 – Disagree, 1 – Strongly Disagree. Read each statement carefully and mark the number that best represents your opinion. | | |
| Variables | **Statements** | **Opinion** |
| Regulatory Influence (RI) | Government regulations encourage our banks to adopt AI for sustainability. |  |
|  | Compliance with sustainability policies influences our AI adoption strategy. |  |
|  | Our bank follows regulatory guidelines for AI-driven green banking solutions. |  |
|  | AI adoption in our bank is influenced by financial regulations on sustainability. |  |
| Perceived Usefulness (PU) | Green AI improves energy efficiency and operational effectiveness in banking. |  |
|  | AI-driven solutions reduce environmental impact while enhancing service quality. |  |
|  | Sustainable AI adoption optimizes financial risk management processes. |  |
|  | The use of AI in sustainability provides long-term benefits for banking operations. |  |
|  | AI-based sustainability tools improve decision-making in financial institutions. |  |
| Perceived Ease of Use (PEOU) | Our bank finds it easy to integrate AI-driven sustainability technologies. |  |
|  | Employees in our bank find Green AI-based banking systems user-friendly. |  |
|  | Green AI implementation does not require extensive technical expertise. |  |
|  | AI-based sustainability systems are compatible with existing banking operations. |  |
| Financial Investment (FI) | Our institution prioritizes financial resources for AI-driven sustainability solutions. |  |
|  | Investments in AI-based sustainability initiatives are increasing annually. |  |
|  | Budget allocations support the Green AI technology implementation in our bank. |  |
|  | Financial considerations play a crucial role in AI sustainability adoption. |  |
| Banking Infrastructure (BI) | Our bank has the necessary technological infrastructure to support AI-based sustainability initiatives. |  |
|  | AI-driven sustainability tools are seamlessly integrated into our bank’s operations. |  |
|  | Our IT infrastructure supports real-time data processing for AI-driven green banking. |  |
|  | Infrastructure limitations do not hinder our ability to adopt AI sustainability solutions. |  |
| Competitive Pressure (CP) | Peer institutions’ adoption of AI influences our bank’s AI sustainability strategies. |  |
|  | Market competition pressures our bank to adopt AI for sustainability compliance. |  |
|  | Industry leaders' AI adoption decisions impact our bank’s strategic sustainability direction. |  |
| Green AI Adoption (GAI) | Our bank actively incorporates AI-driven tools to reduce environmental impact. (e.g., energy-efficient models, carbon-conscious computing). |  |
|  | AI is integrated into our sustainability policies and operational framework. |  |
|  | Our bank prioritizes AI adoption as a key strategy for sustainable development. |  |
| Sustainability Outcomes (SO) | AI implementation in banking has led to lower carbon emissions and energy efficiency improvements. |  |
|  | AI-based sustainability solutions have positively impacted our bank’s ESG compliance. |  |
|  | Green AI adoption has enhanced the bank’s reputation as an environmentally responsible institution. |  |

Table 2A - Standardized Factor Loadings of CFA Constructs

| Construct | Item | Loading | Interpretation |
| --- | --- | --- | --- |
| Regulatory Influence (RI) | RI1 | 0.86 | Acceptable |
|  | RI2 | 0.89 | Acceptable |
|  | RI3 | 0.91 | Excellent |
|  | RI4 | 0.88 | Acceptable |
| Perceived Usefulness (PU) | PU1 | 0.90 | Excellent |
|  | PU2 | 0.88 | Acceptable |
|  | PU3 | 0.91 | Excellent |
|  | PU4 | 0.89 | Acceptable |
|  | PU5 | 0.87 | Acceptable |
| Perceived Ease of Use (PEOU) | PEOU1 | 0.87 | Acceptable |
|  | PEOU2 | 0.89 | Acceptable |
|  | PEOU3 | 0.90 | Excellent |
|  | PEOU4 | 0.88 | Acceptable |
| Financial Investment (FI) | FI1 | 0.90 | Excellent |
|  | FI2 | 0.91 | Excellent |
|  | FI3 | 0.89 | Acceptable |
|  | FI4 | 0.88 | Acceptable |
| Banking Infrastructure (BI) | BI1 | 0.88 | Acceptable |
|  | BI2 | 0.89 | Acceptable |
|  | BI3 | 0.91 | Excellent |
|  | BI4 | 0.87 | Acceptable |
| Competitive Pressure (CP) | CP1 | 0.90 | Excellent |
|  | CP2 | 0.91 | Excellent |
|  | CP3 | 0.88 | Acceptable |
| Green AI Adoption (GAI) | GAI1 | 0.89 | Acceptable |
|  | GAI2 | 0.92 | Excellent |
|  | GAI3 | 0.91 | Excellent |
| Sustainability Outcomes (SO) | SO1 | 0.88 | Acceptable |
|  | SO2 | 0.90 | Excellent |
|  | SO3 | 0.91 | Excellent |

#Source: Author Calculation

All factor loadings exceed the 0.70 threshold, confirming item-level reliability (Hair *et al.,* 2019).

Table 2B - Fornell–Larcker Criterion

| Construct | RI | PU | PEOU | FI | BI | CP | GAI | SO |
| --- | --- | --- | --- | --- | --- | --- | --- | --- |
| RI | **0.809** | 0.61 | 0.54 | 0.49 | 0.57 | 0.45 | 0.62 | 0.55 |
| PU | - | **0.859** | 0.63 | 0.52 | 0.59 | 0.48 | 0.65 | 0.60 |
| PEOU | - | - | **0.878** | 0.50 | 0.53 | 0.46 | 0.61 | 0.54 |
| FI | - | - | - | **0.896** | 0.67 | 0.60 | 0.69 | 0.62 |
| BI | - | - | - | - | **0.898** | 0.66 | 0.72 | 0.65 |
| CP | - | - | - | - | - | **0.837** | 0.59 | 0.53 |
| GAI | - | - | - | - | - | - | **0.899** | 0.78 |
| SO | - | - | - | - | - | - | - | **0.856** |

#Source: Author Calculation

HTMT values for all construct pairs were below the 0.90 thresholds, confirming discriminant validity (Henseler *et al.,* 2015).

Table 2C – Test of Endogeneity using Gaussian Copula

| Predictor | Copula Term Coefficient | Z-Statistic | P-Value | Endogeneity |
| --- | --- | --- | --- | --- |
| FI | 0.032 | 1.12 | 0.262 | No |
| BI | -0.027 | -1.01 | 0.314 | No |
| PU | 0.014 | 0.67 | 0.502 | No |
| PEOU | -0.008 | -0.44 | 0.658 | No |
| RI | 0.019 | 0.73 | 0.467 | No |
| CP | -0.015 | -0.58 | 0.562 | No |

#Source: Author Calculation
